# Altered thermal preference by preoptic estrogen receptor alpha neurons in postpartum females

**DOI:** 10.1016/j.molmet.2025.102108

**Published:** 2025-02-03

**Authors:** Nan Zhang, Meng Yu, Qianru Zhao, Bing Feng, Yue Deng, Jonathan C. Bean, Qingzhuo Liu, Benjamin P. Eappen, Yang He, Kristine M. Conde, Hailan Liu, Yongjie Yang, Longlong Tu, Mengjie Wang, Yongxiang Li, Na Yin, Hesong Liu, Junying Han, Darah Ave Threat, Nathan Xu, Taylor Smiley, Pingwen Xu, Lulu Chen, Tianshu Zeng, Yanlin He, Chunmei Wang

**Affiliations:** 1 Children’s Nutrition Research Center, Department of Pediatrics, Baylor College of Medicine, One Baylor Plaza, Houston, TX, 77030, USA; 2 Department of Endocrinology, Union Hospital, Tongji Medical College, Huazhong University of Science and Technology, Wuhan, Hubei 430022, China; 3 Diabetes and Metabolic Disease Clinical Research Center of Hubei Province, Wuhan, Hubei 430022, China; 4 Hubei Key Laboratory of Metabolic Abnormalities and Vascular Aging, Wuhan, Hubei 430022, China; 5 Hubei Branch of National Center for Clinical Medical Research of Metabolic Diseases, Wuhan, Hubei 430022, China; 6 Pennington Biomedical Research Center, Brain Glycemic and Metabolism Control Department, Louisiana State University, Baton Rouge, LA, 70808, USA; 7 Department of Biological Chemistry, School of Pharmaceutical Sciences, South-central Minzu University, Wuhan, 430074, China; 8 Division of Endocrinology, Diabetes, and Metabolism, Department of Medicine, The University of Illinois at Chicago, Chicago, IL, 60612, USA

**Keywords:** Thermoregulation, Thermosensing, Preoptic area, Estrogen receptor alpha, Reproductive experience

## Abstract

**Objective:**

This study aims to investigate how reproductive experience (RE) alters thermal preference and thermoregulation in female mice, with a focus on estrogen receptor alpha (ERα)-expressing neurons in the preoptic area (POA).

**Methods:**

Thermal preference and body temperature were measured in female mice with and without RE, and virgin female mice with selective deletion of ERα from the POA (ERα^POA^-KO). The number and activity of ERα-expressing POA neurons (ERα^POA^) were assessed using immunohistochemistry and in vitro electrophysiology in response to temperature changes and ERα agonist.

**Results:**

We showed that female mice prefer a cooler environment starting from late pregnancy and persisting long term postpartum. Female mice with RE (>4 weeks post-weaning) displayed lower body temperature and a lower thermal preferred temperature, and lost preference for warm environments (30 °C) but preserved avoidance of cold environments (15 °C). This was associated with a significant decrease in the number of ERα^POA^ neurons. Importantly, virgin female ERα^POA^-KO mice displayed lower thermal preferred temperature and impaired warm preference, mimicking RE mice. We further found that distinct ERα^POA^ subpopulations can be regulated by temperature changes with or without presynaptic blockers, and by ERα agonist. More importantly, RE decreased the number of warm-activated ERα^POA^ neurons and reduced the excitatory effects of warmth and estrogen-ERα signaling, while cold-activated ERα^POA^ neurons were slightly enhanced in female mice with RE.

**Conclusion:**

Our results support that the thermosensing ability and estrogenic effects in ERα^POA^ neurons are regulated by reproductive experience, altering thermal preference.

## Highlights

• Reproductive experience or loss of ERα^POA^ drives females to a cooler environment.
• ERα^POA^ neurons are heterogeneous, and sense temperature increase or decrease.
• Subpopulations of ERα^POA^ neurons can be directly activated by warmth or cold.
• Subpopulations of ERα^POA^ neurons can be activated or inhibited by E2-ERα signaling.
• Reproductive experience modulates the E2 and warm excitation of ERα^POA^ neurons.

## 1 Introduction

Females experience significant metabolic adaptations during pregnancy and lactation to support extra nutrition for the development and growth of pups, including increased feeding and suppressed energy expenditure [[1], [2], [3]]. Female brain is significantly remodeled during pregnancy and lactation to accommodate reproduction and motherhood, as well as these metabolic adaptations, and some of the adaptations persist long-term postpartum [[4], [5], [6], [7], [8]]. Three-quarters of the maternal population maintain an increased body weight after pregnancy even one year after delivery, and this postpartum body weight retention is known to be a potential risk for obesity in women [[9], [10], [11]]. Consistently, female mice with reproductive experience (RE) maintain an increased body weight even after 2 months from weaning, potentially contributed by a strong increasing trend of food intake and decreased physical activity [12]. Energy expenditure includes both physical activity and thermoregulation. Animals decrease energy expenditure and body temperature in response to elevating environmental temperature and do the opposite in response to cold [[13], [14], [15]]. However, the thermal regulation during and after reproduction is understudied. In both humans and mice, body temperature increases during early pregnancy, drops to normal temperature during late pregnancy, and then goes up again during lactation [[16], [17], [18]]. Mammals prefer to stay within a range of temperature close to thermoneutrality, referred as thermal comfortable temperature or preferred temperature. Despite a normal body temperature during late pregnancy, the thermoneutral temperature decreases in human [17], and sows prefer a cooler temperature than non-pregnant sows [19]. However, the long-term impact of RE on thermosensing, thermoregulation and their underlying mechanisms are unclear.

Mammals maintain stable core temperature in a temperature-changing environment through thermoregulation. When ambient temperature increases above thermoneutral temperature, warm sensing neurons are activated, and body temperature is decreased via suppression of thermogenesis, facilitation of heat loss or cold-seeking behavior. On the other hand, when ambient temperature drops below thermoneutral temperature, cold-sensing neurons are activated, and body temperature is increased via opposite actions [[13], [14], [15]]. These thermal defensive behaviors help animals to combat thermal stresses and maintain thermostasis. Thermosensing and thermoregulating neurons are located in many hypothalamic regions, including the preoptic area (POA) [14,15,[20], [21], [22], [23], [24], [25], [26], [27], [28], [29], [30], [31], [32], [33], [34]], the lateral hypothalamus (LH) [27,35], the dorsomedial hypothalamus (DMH) [36], and the ventromedial hypothalamus (VMH) [37]. In addition to thermal defensive behaviors, warm- or cold-seeking behaviors are also driven by valence produced by ambient temperature changes. An extreme cold environment produces a negative valence associated with cold-activated neurons [15,38]. In an environment with temperature below or around thermoneutral temperature, increase of temperature will activate warm-sensing neurons to produce a warmth reward [38]. Consistently, activation of warm-activated neurons in the POA produces a positive valence specifically at temperatures below thermoneutrality [15], and this warmth reward may drive the mice to move toward a warm environment, potentially independent of cold defensive behavior [[13], [14], [15]]. However, in a hot environment (e.g., >37 °C), increase of temperature can produce a hotness-punishing effect [35]. The negative feelings produced by extreme cold and hot together with the warm reward will drive humans or animals to move to an environment with a temperature range where they feel most comfortable, and this temperature range is defined as preferred temperature.

The POA is critical for temperature sensing and thermoregulation. A large body of evidence showed that POA is rich in warm-sensing neurons [[30], [31], [32]], and activation of these neurons can reduce body temperature [14,15,33,34]. Recent studies also demonstrated cold-sensing neurons in the POA [22], and activation of a population of POA neurons can increase body temperature [24,33]. POA neurons are significantly remodeled during pregnancy and lactation [39,40]. Gonadal hormones, including 17β-estradiol (E2), are significant contributors to shaping the maternal brain, including the POA, a major brain region responsible for maternal behavior [41]. E2 can also modulate the thermoregulation function of POA neurons. Evidence has shown that the decline of E2 signaling and the modulation of POA neurons during menopause contribute to menopausal hot flashes [25,26,42]. Estrogen receptor alpha (ERα) is highly expressed in the POA [43,44], and ERα-expressing POA neurons (ERα^POA^) are important for thermoregulation in females [34,42]. A group of ERα^POA^ neurons can be activated by an increase in temperature and activation of ERα^POA^ neurons dramatically reduces body temperature in mice [34]. In cycling females, E2 peaks at proestrus and drops during estrus, and then stays low till proestrus again [45]. However, a recent study reported an unexpected E2 peak during diestrus in mice [46]. E2 levels rise during late pregnancy, drop during lactation, and then gradually recover after the weaning of pups [[47], [48], [49]]. Importantly, female RE rats displayed lower serum E2 levels at 2 weeks after the weaning of pups, suggesting prolonged postpartum modulation of estrogenic effects [50]. These findings clearly indicate an essential role of estrogen/ERα^POA^ signaling in regulating temperature homeostasis. However, the temperature-sensing ability of ERα^POA^ neurons and their modulations by RE are not well defined.

Here we reported lower body temperature, a reduced thermal preferred temperature and impaired warm preference associated with a decreased number of ERα^POA^ neurons in RE mice compared to virgin female mice. Then we used mouse models with loss of ERα specifically in POA neurons to investigate the contribution of ERα^POA^ neurons to temperature-sensing behaviors in female mice. Further, we characterized subpopulations of ERα^POA^ neurons responding to temperature changes with or without presynaptic blockers. Finally, we investigated how RE modulated the temperature-sensing abilities and estrogenic effects in ERα^POA^ neurons.

## 2 Results

### 2.1 Reproductive experience impairs warmth-seeking behavior in female mice

**Figure 1:**
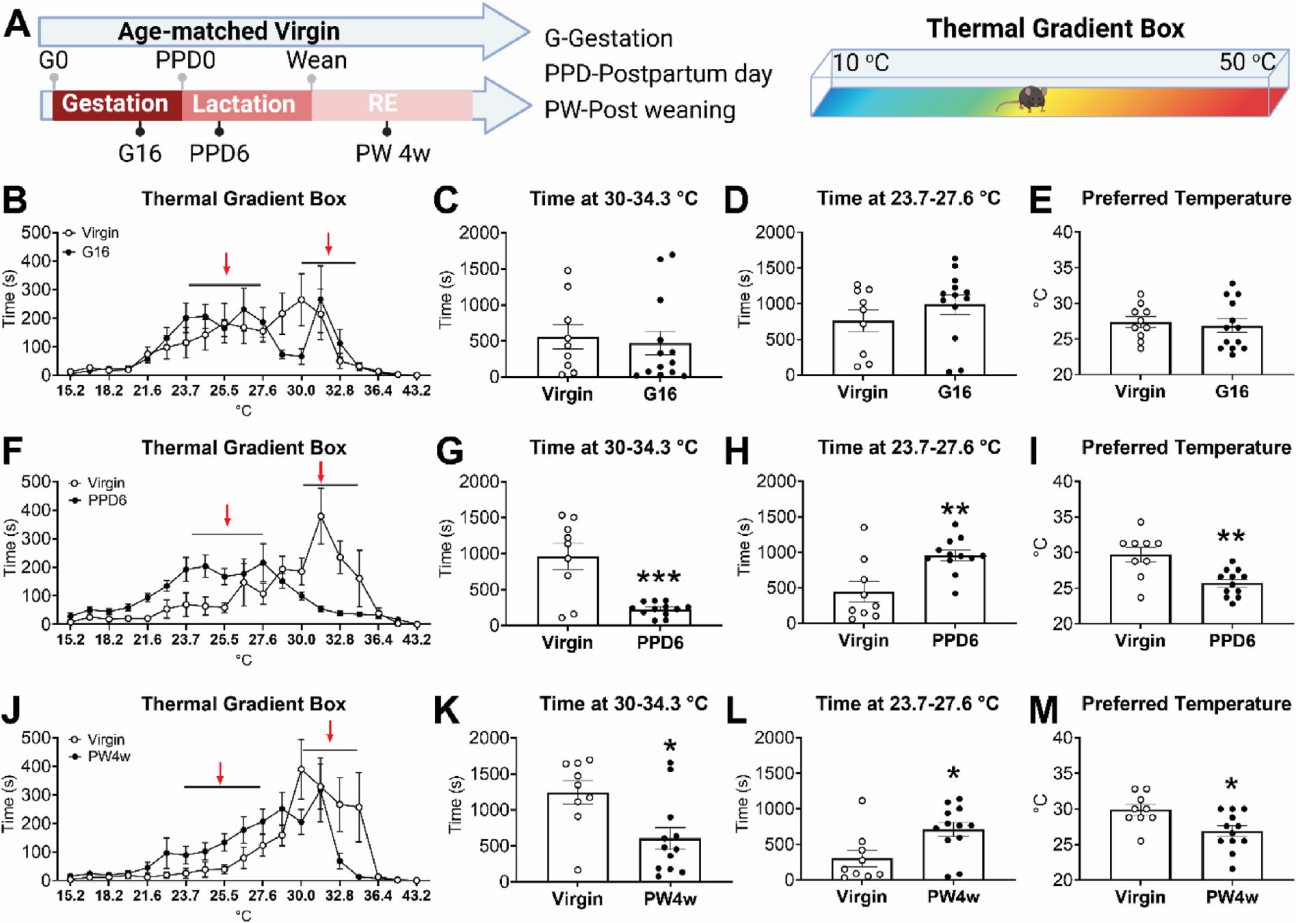
**Modulation of thermoneutral temperature during and after reproduction.** (A) Schematic design of measurements for thermal preference temperature in mice during and after reproduction (left), using a thermal gradient box with a gradient of ambient temperature from 10 to 50 °C (right). (B–D) Time that late pregnant mice on gestation day 16 and their age-matched virgin female mice spent in a thermogradient box (B), and at 30–34.3 °C (C) or 23.7–27.6 °C (D). (E) The preferred temperature that late pregnant mice on gestation day 16 and their age-matched virgin female mice spent in a thermal gradient box. (F–H) Time that lactating mice on postpartum day 6 (PPD6) and their age-matched virgin female mice spent in a thermal gradient box (F), and at 30–34.3 °C (G) or 23.7–27.6 °C (H). (I) The preferred temperature that PPD6 lactating mice and their age-matched virgin female mice spent the longest time in a thermal gradient box. (J–L) Time that RE mice (4 weeks after the weaning of pups) and their age-matched virgin female mice spent in a thermal gradient box (J), and at 30–34.3 °C (K) or 23.7–27.6 °C (L). (M) The preferred temperature that RE mice and their age-matched virgin female mice spent in a thermal gradient box. Red arrows (B, F and J) indicate the areas displaying the most dramatic differences. Data are presented as mean ± SEM or with individual data points. ∗, ∗∗, *P* < 0.05, or 0.01 in two-tailed unpaired t-tests. *N* = 9–13 for B-E. *N* = 9–12 for F-M. (For interpretation of the references to colour in this figure legend, the reader is referred to the Web version of this article.)

Females experience significant metabolic changes during and after reproduction. Previous reports indicate altered body temperature and thermal preference during late pregnancy and nursing, and metabolic changes long-term postpartum [[16], [17], [18]]. To explore the mechanism, we compared the thermoregulation of C57BL/6 wild type (WT) female mice between virgin and three snapshots of reproduction (Figure 1A): late pregnancy (gestation day 16th, G16), lactation (postpartum day 6th PPD6) and reproduction experienced (RE, 4 weeks post-weaning, PW4w). Consistently, compared to age-matched virgin female mice, body temperature of reproductive mice increased during early pregnancy but dropped back during late pregnancy, and then went up again during lactation (Supplemental Fig. 1A). Interestingly, we found that body temperature was decreasing post lactation and displayed a decreasing trend in PW5w RE mice, which resulted in lower body temperature even 4 months after weaning (Supplemental Figs. 1B–C), suggesting a complex modulation of body temperature during different stages of reproduction.

We then used a thermal gradient box to measure the thermal preferred temperature as an estimation of thermoneutral temperature (Figure 1A), i.e., the temperature range that mice preferred to stay in when they were allowed to explore an environment with variable ambient temperature (10–50 °C). Virgin female mice preferred to stay at 28–34 °C at all tested ages (10–20 weeks, Figure 1). Similar to the decreased thermoneutral temperature in sows and humans during late pregnancy [17,19], G16 mice, representing late pregnancy, spent less time in the thermal neutral zone around 30 °C, but without significant changes at the warm 30–34.3 °C area or at cooler area 23.7–27.6 °C area, resulting in unchanged preferred temperature. However, they decreased physical activity as indicated by decreasing travel distance and inactive time during the test (Figure 1B–E, Supplemental Fig. 1D and 1H). Interestingly, the preference for a cooler environment was enhanced during lactation but together with increased physical activity (Figure 1F–I, Supplemental Fig. 1E and I). Lactating mice on PPD6 stayed much longer in a cooler environment (23.7–27.6 °C), resulting in a correspondingly shorter time at a warm environment (30–34.3 °C). Consequently, the thermal preferred temperature was also significantly decreased in lactating mice (Figure 1F–I). Importantly, we found that the lactational drop of thermal preferred temperature persisted long-term postpartum, as PW4w RE mice still preferred to stay in a cooler environment with reduced thermal comfortable temperature (Figure 1J-M). However, physical activity was recovered at PW4w (Supplemental Figs. 1F and J). In virgin female mice, the time spent in the warm area was correlated negatively with travel distance and positively with the inactive time. However, these correlations were uncoupled in G16 and PPD6 dams and then gradually recovered on PW4w (Supplemental Fig. 1G and 1K). Notably, there were 2 outliers in G16 dams displaying similar pattern to virgin female mice with significantly longer time in the warm area, implying G16 as an initial stage of the modification (Figure 1B–D, Supplemental Fig. 1G and 1K). One study reported that large size but not middle size pigs prefer cooler environment than small size pigs, suggesting the impact of body weight on thermal preference [51]. However, pregnant mice had the highest body weight but lactating female mice displayed the most profound cool preference, and the body weight was comparable between RE mice and age matched virgin female mice (Supplemental Fig. 2A). This result does not support the shift of thermal preference as secondary to body weight change. We further analyzed the correlation between body weight and the preferred temperature, or the time spent in each zone. Interestingly, body weight of virgin female mice was positively correlated with warm preference and negatively correlated with cool preference, especially at older age (Supplemental Fig. 2B-M). However, the positive correlation between body weight and warm preference, but not the negative correlation between body weight and cool preference, was uncoupled in RE mice (Supplemental Fig. 2L and 2M). Importantly, both PPD6 and RE mice displayed distinct thermal preference with the adjustment of body weight (Supplemental Fig. 2H-M). These results suggested a chronic alteration of thermal preference attributed to reproduction, independent of changes in body temperature, physical activities and body weight.

**Figure 2:**
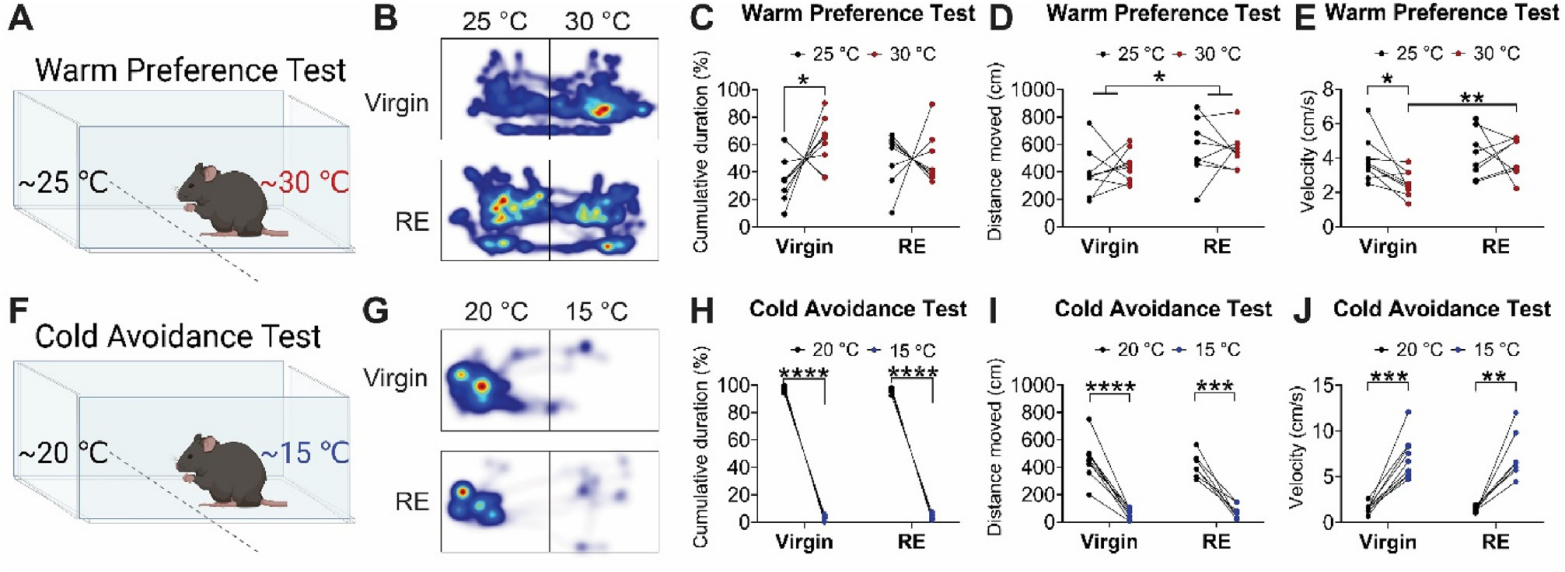
**RE mice lost preference to a warm environment.** (A) Schematic design of warm preference test with one side set to 25 °C and the other side to 30 °C. RE mice (7 months after weaning) or age-matched virgin female mice were allowed to freely explore the two sides for 5 min. (B) Heat map of the time that mice spent in the two sides of the test box. (C–E) Percentage of duration (C), traveling distance (D) and velocity (E) that each mouse explored in each side of the box. Data are presented as individual data points. ∗ and ∗∗, *P* < 0.05 or 0.01 in two-tailed paired t-tests between 25 °C side vs 30 °C side (C and E) or two-tailed unpaired t-tests between virgin vs RE (D and E). *N* = 7–8. (F) Schematic design of cold avoidance test with one side set to 15 °C and the other side to 20 °C. (G) Heat map of the time that mice spent in the two sides of the test box. (H–J) Percentage of duration (H), traveling distance (I) and velocity (J) that each mouse explored in each side of the box. Data are presented as individual data points. ∗∗, ∗∗∗, ∗∗∗∗, *P* < 0.01, 0.001 or 0.0001 in two-tailed paired t-tests between 20 °C side vs 15 °C side (H–J). *N* = 7–8.

To further investigate the altered thermal preference in RE mice, we used the warm preference or cold avoidance tests to compare temperature preference of RE female mice (7 months post-weaning) and age-matched virgin female mice. In the warm preference test, we utilized a home cage with one side heated to around 30 °C, approximately at thermoneutrality for mice, and the other side was kept at around 25 °C, approximately at regular mouse housing temperature (Figure 2A). When allowed to freely explore, WT virgin female mice preferred to stay in the warm side, displaying a warm preference. However, age-matched RE female mice lost this warm preference, spending comparable time on each side (Figure 2B–C). Interestingly, there was no difference in the total distance traveled between 30 °C and 25 °C sides in either virgin or RE mice (Figure 2D). Virgin, but not RE, mice reduced travel velocity on their preferred 30 °C side, suggesting a potential settling down behavior or resting status (Figure 2E). On the other hand, RE mice displayed higher total travel distance during the entire testing period and higher velocity on the 30 °C side compared to virgin female mice (Figure 2D–E).

In the cold avoidance test, one side of the cage was cooled to around 15 °C, and the other side was kept at about 20 °C (Figure 2F). Virgin female mice displayed a robust cold avoidance behavior, spending the majority of time on the 20 °C side. RE mice showed the same cold avoidance behavior as virgin female mice (Figure 2G–H). Interestingly, both RE and virgin mice increased travel velocity, but reduced total travel distance on the 15 °C cold side (Figure 2I–J), indicating a strong escape behavior.

Together, these results indicate that RE mice lost warm preference associated with a lower thermal preferred temperature.

### 2.2 Loss of ERα in the POA impairs warmth-seeking behavior in virgin female mice

E2 is a reproductive and metabolic hormone [[52], [53], [54], [55], [56], [57], [58], [59]], and E2 signaling is significantly modulated during reproduction [[60], [61], [62]]. Body temperature is generally higher at estrus, following E2 peaks at diestrus and proestrus [45,46,63]. Vehicle treated ovariectomized (OVX-V) rats prefer an environment 4 °C cooler than E2 treated ovariectomized (OVX-E) rats [64], supporting the thermoregulatory role of E2. We checked the estrous cycles of virgin female mice on the day of thermogradient box test (control for PPD6), and then replotted the data based on their estrous and diestrus groups. We found that estrus mice displayed typical warm preference with peak at 31.3 °C. However, diestrus virgin mice displayed altered warm preference without an obvious peak area. Diestrus virgin mice spent comparable time across a broad range of temperature zones, resulting in a relatively flat curve. Interestingly, diestrus virgin mice showed random time spent in the cooler environment (23.7–27.6 °C) and random preferred temperature with significantly bigger variations than estrous mice, despite the mean values were not significantly different between these two groups (Supplemental Fig. 3). These results suggest that the innate status changed by E2 signaling, rather than instant E2 level, plays a critical role for the maintenance of warm preference in female mice.

**Figure 3:**
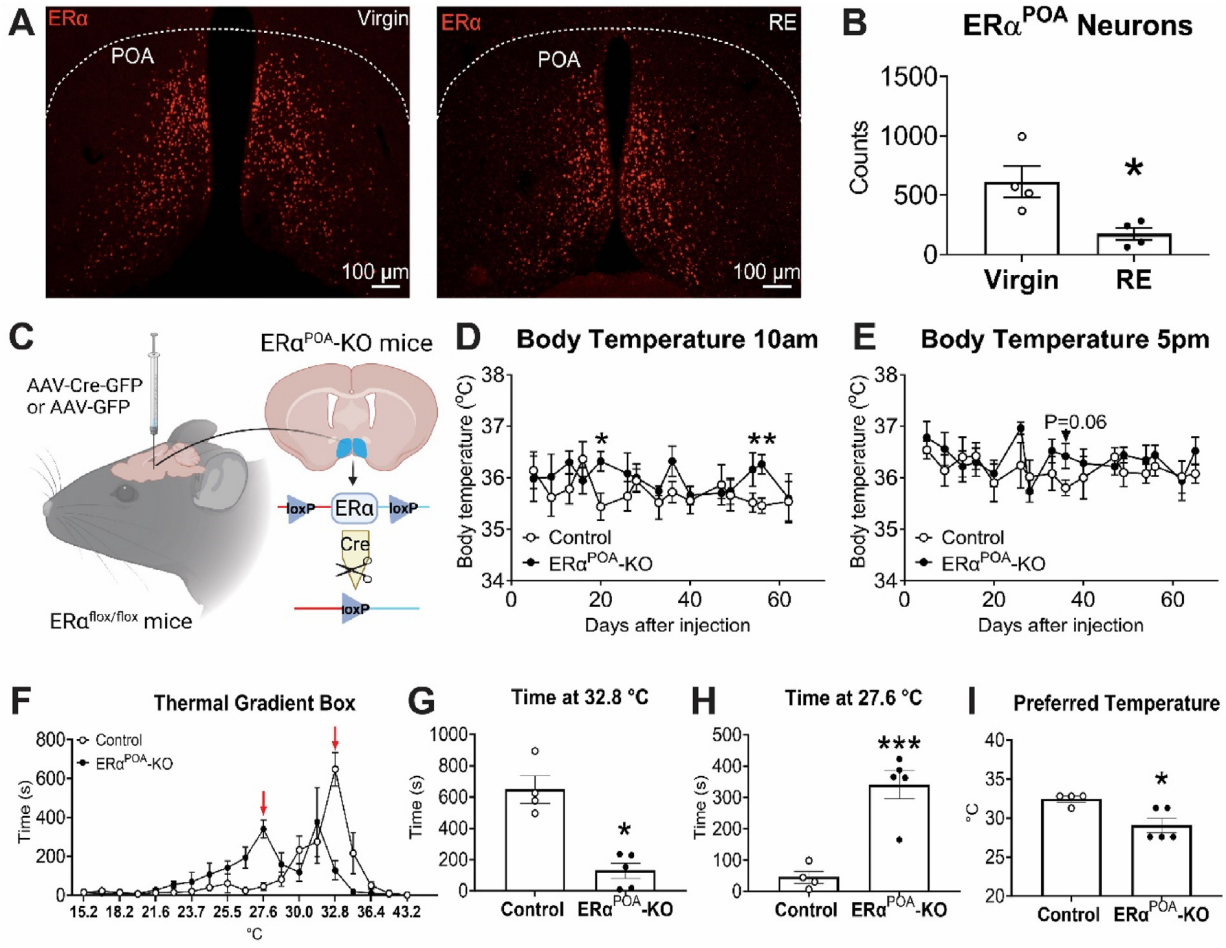
**RE mice displayed reduced number of ERα**^**POA**^**neurons and virgin female ERα**^**POA**^**-KO mice lost warm preference.** (A–C) Immunostaining for ERα (A) and quantification of ERα neurons (B) in the POA of RE (6–12 days after weaning) and age-matched virgin female mice at the fed and diestrus phase. Data are presented as mean ± SEM or with individual data points. ∗, *P* < 0.05 in two-tailed unpaired t-tests. *N* = 3–4. (C) Schematic design of virus injections in ERα^flox/flox^ mice to delete ERα in POA neurons. (D–E) Body temperature (E) of virgin ERα^POA^-KO mice and control female mice measured at 10am (D) or 5pm (E). (F) Time that mice spent in a thermogradient box with ambient temperature from 10 to 50 °C. (G–H) Time that mice spent at 32.8 °C (G) and 27.6 °C area (H). (I) The preferred temperature that mice spent in a thermogradient box. Red arrows (F) indicate the areas displaying the most dramatic differences. Data are presented as mean ± SEM or with individual data points. ∗ and ∗∗∗, *P* < 0.05 and 0.001 in two-tailed unpaired t-tests. *N* = 4–5. (For interpretation of the references to colour in this figure legend, the reader is referred to the Web version of this article.)

The metabolic effects of E2 are mediated primarily by ERα, especially in hypothalamic neurons [42,[64], [65], [66], [67], [68]]. Published single cell RNA-Seq (scRNA-Seq) data [43,44] indicate high expression of ERα but very low ERβ and undetectable G protein-coupled estrogen receptor (GPER) in the POA, suggesting that estrogenic effects in the POA are primarily mediated by ERα. Here we compared the expression of hypothalamic ERα between RE mice 1–2 weeks after weaning and age-matched virgin female mice, and we found a significant decrease of ERα neuron numbers in the POA (Figure 3A–B), with no change in the ventromedial hypothalamic nucleus (VMH) but a trend of decrease in the arcuate nucleus of the hypothalamus (ARH) (Supplemental Figs. 4A–C), despite a previous report showed that ERα expression in the ARH is not changed by RE 1 month post-weaning [12]. These results suggest a potential role of ERα in the POA related to changed behavior seen in RE mice.

To determine whether the reduction of ERα in POA neurons contributes to the altered thermal preference in RE mice, we injected an AAV-Cre-GFP virus into the POA of virgin female ERα^flox/flox^ mice to generate mice with ERα selectively deleted from POA neurons (ERα^POA^-KO). Simultaneously, littermate virgin female ERα^flox/flox^ mice that received an AAV-GFP virus were used as control (Figure 3C, Supplemental Fig. 4D). Body weight and food intake were comparable between control and ERα^POA^-KO virgin female mice (Supplemental Figs. 4E–F). Interestingly, the body temperature of the ERα^POA^-KO mice displayed a general increasing trend with a few transient significant increases, especially in the morning (Figure 3D–E). Using a thermal gradient box, we found that ERα^POA^-KO virgin female mice spent significantly more time in a lower temperature area (27.6 °C) and less time in a higher temperature area (32.8 °C) than control virgin female mice, resulting in two peaks. Consistently, ERα^POA^-KO virgin female mice displayed lower preferred ambient temperature (Figure 3F–I). Notably, physical activity was not changed in ERα^POA^-KO mice (Supplemental Figs. 4G–H). These results indicate that loss of ERα from the POA in virgin female mice reduces the thermal preferred temperature or warm preference, mimicking lactating and RE female mice.

### 2.3 ERα^POA^ neurons response to temperature changes from both external environment and internal brain

**Figure 4:**
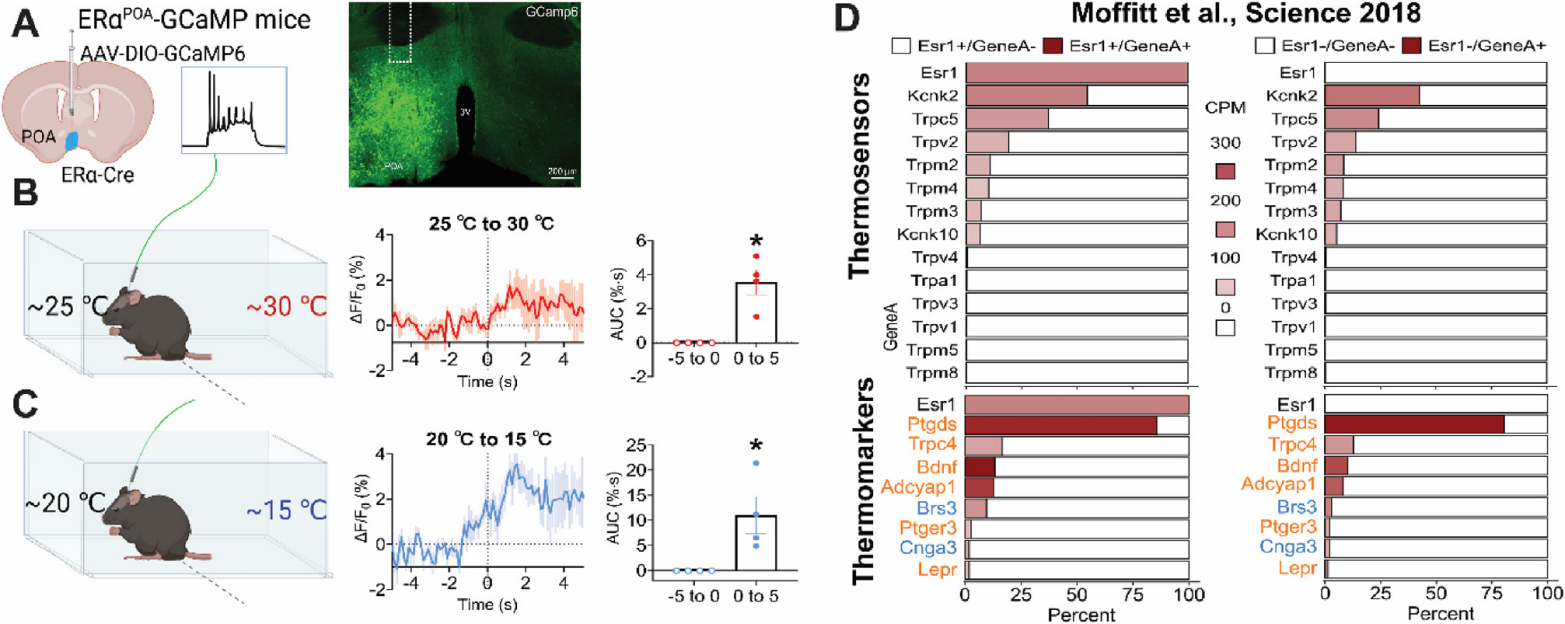
**ERα**^**POA**^**neurons sensed temperature changes from the environment and brain.** (A) Schematic design of virus injections (left) and validation (right) in the ERα-Cre mice to generate virgin ERα^POA^-GCaMP6 female mice and fiber photometry recording of ERα^POA^ neuron activities at temperature-changing environments. (B) ERα^POA^ neurons were activated when mice travelled from 25 °C side to 30 °C side as indicated by increased GCaMP6 signal in the middle panel. Time 0 is defined as mice travelling across the line between the two sides. The right panel calculated the area under the curve based on 5 s before and after the cross. (C) ERα^POA^ neurons were activated when mice travelled from 20 °C side to 15 °C side as indicated by increased GCaMP6 signal in the middle panel. Time 0 is defined as mice travelling across the line between the two sides. The right panel calculated the area under the curve based on 5 s before and after the cross. Data are presented as mean ± SEM. ∗, *P* < 0.05 in two-tailed unpaired t-tests (Mann Whitney test). *N* = 4 mice, 8–10 trials in total. (D) The expression of genes encoding thermosensors and thermomarkers in ERα^POA^ (left) and non-ERα^POA^ (right) neurons by a secondary analysis of published scRNA-Seq data [43]. CPM, counts per million. The color intensity indicates the abundance of each gene in ERα^POA^ neurons, while the width of the bar represents the percent expressing of each gene. (For interpretation of the references to colour in this figure legend, the reader is referred to the Web version of this article.)

To determine whether the activity dynamics of ERα^POA^ neurons can respond to changes in ambient temperature, we used fiber photometry to record the activity of the whole ERα^POA^ population in freely moving virgin female mice. We injected Cre-dependent AAV-DIO-GCaMP6 virus into the POA of ERα-Cre mice to express GCaMP6 protein specifically in all ERα^POA^ neurons (ERα^POA^-GCaMP6). Then we set up an optic fiber onto the POA to record the activity of ERα^POA^ neurons (Figure 4A). We found that the activity of ERα^POA^ neurons increased when mice entered the 30 °C side from the 25 °C side (Figure 4B). Interestingly, the activity of ERα^POA^ neurons also increased when mice entered the 15 °C side from the 20 °C side (Figure 4C), with higher amplitude than 30 °C excitation. It is potentially because 25–30 °C is within the thermal comfortable zone of mice, and mice are used to this temperature range that the responses are mild. However, 15 °C is too cold for mice, thus cold can induce higher response. These results indicate that ERα^POA^ neurons can respond to changes in ambient temperature *in vivo*.

The acute response in ERα^POA^-GCaMP6 mice should be sensed by skin thermosensors and then sent back to the brain via a circuit back to the POA without changing body temperature or brain temperature. In addition to this peripheral mechanism, temperatures inside the brain, especially the POA, tightly fluctuate with body temperature when exposed to different environmental temperatures for long time [69]. Thermosensing can also be achieved by thermosensors in the thermosensing neurons directly [33,69]. ERα^POA^ neurons have been reported to sense brain temperature increase [34]. To find out how ERα^POA^ neurons sense brain temperature change, we re-analyzed published single cell RNA-Seq (scRNA-Seq) data [43,44] of POA neurons. We found abundant expression of thermosensors in ERα^POA^ neurons, including many members of Transient Receptor Potential (TRP) channels and cold sensors two-pore potassium channels (K2Ps) [[70], [71], [72], [73], [74]] (Figure 4D and Supplemental Fig. 5A). Interestingly, the most abundant sensors including transient receptor potential cation channel 5 (Trpc5) and potassium two pore domain channel subfamily K member 2 (Kcnk2) are both cold sensors [[70], [71], [72], [73],^75^], while transient receptor potential cation channel subfamily M member 2, 3, and 4 (Trpm2, 3, 4) are warm sensors responsible for different warm temperature ranges [70,76]. In addition, we found that portions of ERα^POA^ neurons also co-expressed many identified warm- or cold-sensing neuron markers, including transient receptor potential cation channel 4 (Trpc4), leptin receptors (LepR) [77], brain-derived neurotrophic factor (Bdnf) and pituitary adenylate-cyclase-activating polypeptide (Adcyap1) [14,15], lipocalin-type prostaglandin-D synthase (Ptgds) [33], EP3 prostaglandin receptor (Ptger3) [23] as warm-sensing markers, and bombesin receptor subtype 3 (Brs3) and cyclic nucleotide gated channel subunit alpha 3 (Cnga3) [22,24,33] as cold-sensing neurons (Figure 4D and Supplemental Fig. 5A). Notably, despite not being a direct warm thermoreceptor, Trpc4 is essential for internal warm sensing in the POA [69]. Interestingly, the expression of these temperature sensors and neurons markers are similar between ERα^POA^ neurons and non-ERα^POA^ neurons, suggesting that ERα^POA^ neurons represent a subpopulation of each neuron type (expressing each thermosensor or thermomarker) that is regulated by E2-ERα^POA^ signaling. Despite that the expression profiles of these thermosensors were not altered between OVX-V and OVX-E female mice, OVX-V mice displayed a decrease of warm-sensing neuron markers like Trpc4 and Ptgds, but an obvious increase of cold-sensing neuron marker Cnga3 (Supplemental Fig. 5A), suggesting a potential estrogenic role in the thermal regulation of ERα^POA^ neurons.

### 2.4 Warm- and cold-activated ERα^POA^ neurons are modulated in RE mice

**Figure 5:**
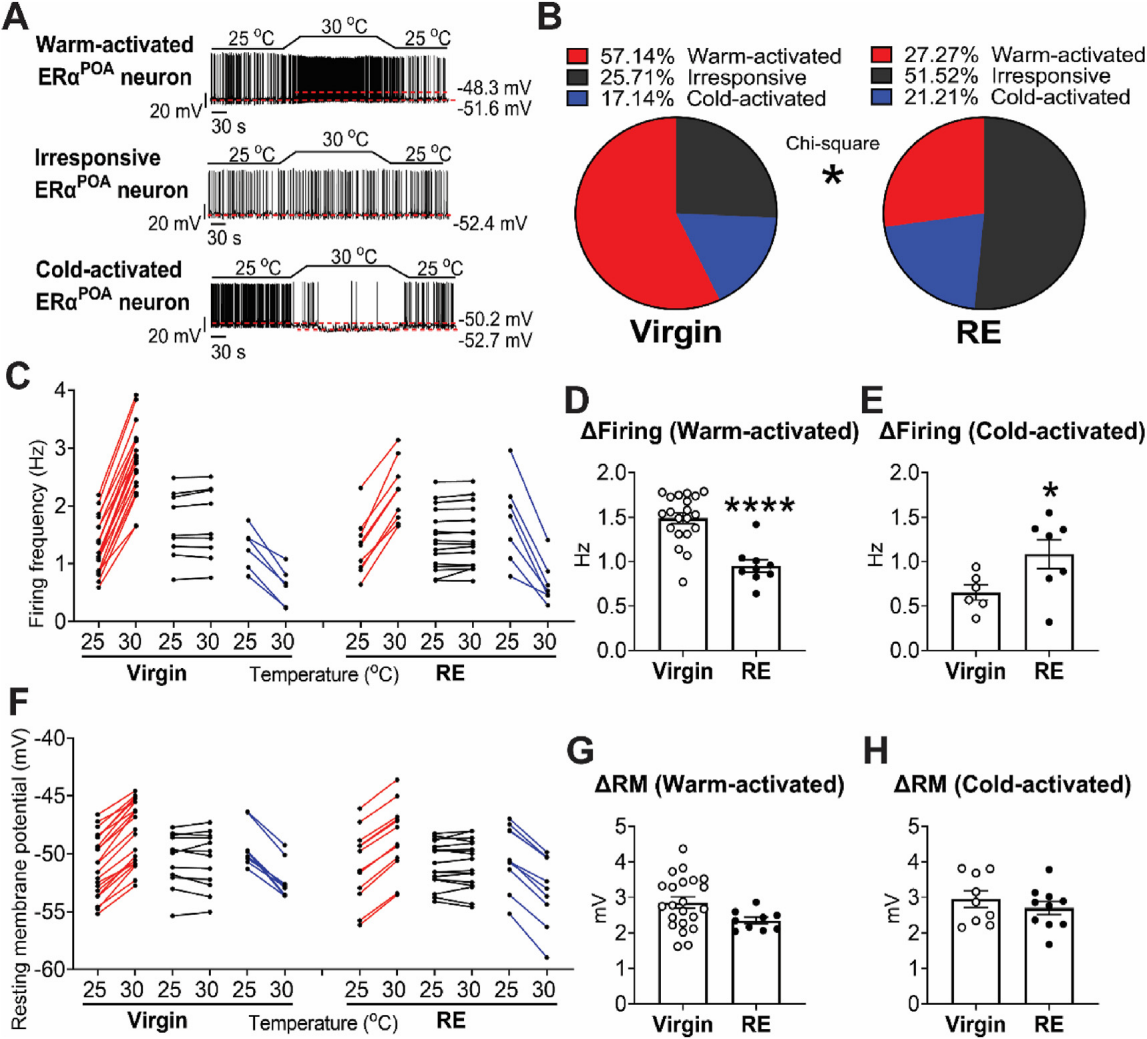
**ERα**^**POA**^**neurons were regulated by changing temperature and RE.** (A) Representative traces of an ERα^POA^ neuron excited (up), unresponsive (middle) or inhibited (down) by temperature increasing, defined as warm-activated, unresponsive or cold-activated ERα^POA^ neuron. Warm activation or inhibition was defined as either >20% increases or decreases in firing rate or by > 2 mV depolarization or hyperpolarization. (B) Percentage of warm-activated, unresponsive or cold-activated ERα^POA^ neurons in age-matched virgin (4–5 months of age) and RE female mice (1 month post-weaning). (C–E) Spontaneous firing rate (C), increases of firing rate in warm-activated ERα^POA^ neurons when temperature went up from 25 °C to 30 °C (D) and in cold-activated ERα^POA^ neurons when temperature went down from 30 °C to 25 °C (E). (F–H) Resting membrane potential (F), depolarization in warm-activated ERα^POA^ neurons when temperature went up from 25 °C to 30 °C (G) and in cold-activated ERα^POA^ neurons when temperature went down from 30 °C to 25 °C (H). Data are presented as part of whole bar graphs (B) or mean ± SEM and/or individual data points (C–H). ∗, *P* < 0.05 in χ2 test, Virgin vs RE (B). ∗ and ∗∗∗∗, *P* < 0.05 or 0.0001 in two-tailed unpaired t-tests Virgin vs RE (D, E and L). *N* = 12–18 neurons from 3 mice/group.

The fiber photometry recorded the activity of the ERα^POA^ neuron population as a whole, but it could not distinguish the subpopulations that may differentially respond to cold or warm temperature. To assess the responses of individual ERα^POA^ neurons, we used an ERα-ZsGreen transgenic mouse line in which ERα^POA^ neurons were labelled with ZsGreen [78], and we recorded the temperature-sensing ability of individual ERα^POA^ neurons using *ex vivo* slice electrophysiology (Supplemental Fig. 5B). Consistent with a previous report [34], we found that a subset of ERα^POA^ neurons displayed increased action potential firing frequency and depolarized resting membrane potential when the bath temperature went up from 25 °C to 30 °C, and then recovered when the bath temperature went back to 25 °C; these neurons were defined as warm-activated neurons. On the other hand, a distinct subset of ERα^POA^ neurons responded oppositely. They displayed decreased action potential firing and hyperpolarized resting membrane potential with increasing bath temperature and recovered with decreasing temperature; these neurons were defined as cold-activated neurons. Another subset of ERα^POA^ neurons was unaffected by temperature fluctuations, defined as irresponsive ERα^POA^ neurons (Figure 5A,C and F). Together, these results further support that subpopulations of ERα^POA^ neurons can sense warm and cold temperatures, respectively.

To further explore whether the temperature sensing abilities of ERα^POA^ neurons were modulated by reproductive experience, we compared the number and activity of warm- or cold-activated ERα^POA^ neurons between virgin and RE mice. In virgin female mice, 57.14% of ERα^POA^ neurons were warm-activated, and 17.14% were cold-activated, with 25.71% being unresponsive neurons. In contrast, RE mice displayed decreased warm-activated neurons, a slight increase in cold-activated neurons and doubled unresponsive neurons (Figure 5B,C and F). In addition, warm-induced increase of action potential firing frequency was significantly decreased and the depolarized resting membrane potential had a decreasing trend in warm-activated ERα^POA^ neurons from RE mice compared to those from virgin female mice (Figure 5D,G); in contrast, cold-induced increase of action potential firing frequency was increased in cold-activated ERα^POA^ neurons from RE mice compared to those from virgin female mice, without changes in the resting membrane potential (Figure 5E,H). Together, these results indicate that RE impaired warm-excitation but slightly enhanced cold-excitation of ERα^POA^ neurons in female mice.

To further test whether the temperature sensing abilities of ERα^POA^ neurons are intrinsic, we recorded ERα^POA^ neurons again using the same protocol, but with presynaptic blockers to block any peripheral inputs (Supplemental Fig. 6A). ERα^POA^ neurons could still be activated by temperature increase or decrease (Supplemental Fig. 6B), and the populations of the warm- and cold-activated and unresponsive neurons were similar in virgin female mice, and displayed similar changes in RE mice with impaired warm-excitation and enhanced cold-excitation abilities (Supplemental Figs. 6C–H). In other words, presynaptic blockers did not change the thermosensing ability of ERα^POA^ neurons and their modulation by reproductive experience, confirming their intrinsic thermosensing ability via thermosensors expressed in ERα^POA^ neurons.

### 2.5 E2 responsiveness of ERα^POA^ neurons is modulated in RE mice

**Figure 6:**
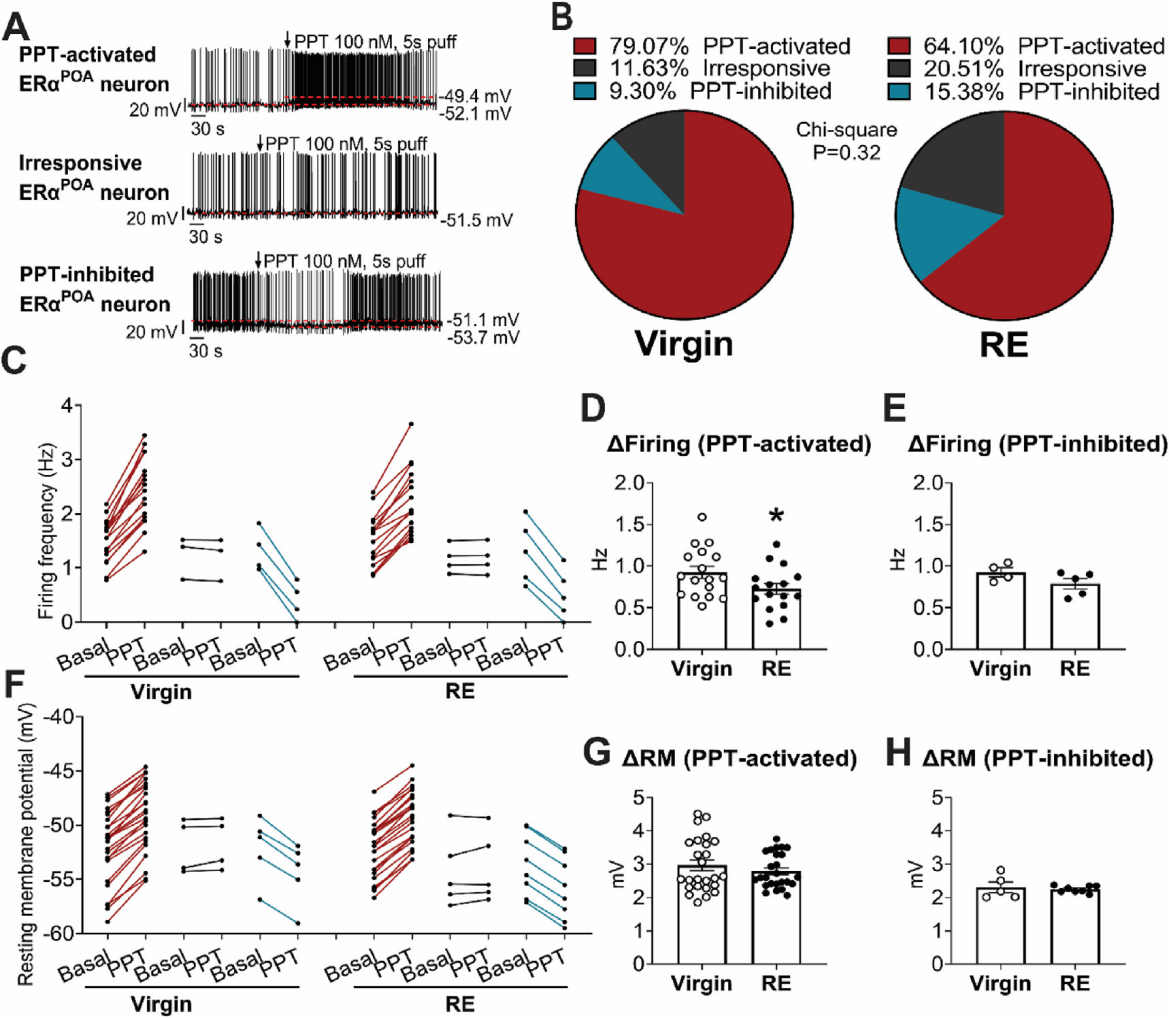
**ERα**^**POA**^**neurons were regulated by E2 and RE.** (A) Representative traces of ERα^vlVMH^ neurons excited (up), unresponsive (middle) or inhibited (down) by PPT. (B) Percentage of ERα^POA^ neurons regulated by PPT in age-matched virgin (4–5 months of age) and RE female mice (1-month post-weaning). PPT-induced activation or inhibition was defined as either >20% increases or decreases in firing rate or by > 2 mV depolarization or hyperpolarization. (C–E) Spontaneous firing rate (C), increases of firing rate in PPT-activated ERα^POA^ neurons (D) and decreases of firing rate in PPT-inhibited ERα^POA^ neurons (E). (F–H) Resting membrane potential (F), depolarization in PPT-activated ERα^POA^ neurons (G) and hyperpolarization in PPT-inhibited ERα^POA^ neurons (H). Data are presented as part of whole bar graphs (B) or mean ± SEM and/or individual data points (C–H). ∗, *P* < 0.05 in two-tailed unpaired t-tests Virgin vs RE (D). *N* = 12–18 neurons from 3 mice/group.

ERα in the POA can act as a nuclear transcriptional factor to mediate the thermoregulatory role of E2 through the classic nuclear-initiated genetic effect. This is evidenced by the gene profile changes of ERα^POA^ neurons by brain specific deletion of ERα [42], or loss/gain of E2 in virgin female mice (Supplemental Fig. 5). In addition, E2 also binds to ERα located on the membrane, which triggers an acute signaling pathway and quickly increases neural firing activity [[79], [80], [81], [82]]. To test this membrane-initiated quick response of estrogenic effect, we examined the responses of ERα^POA^ neurons to propyl pyrazole triol (PPT), a selective ERα agonist [82] (Figure 6A). In virgin female mice, 79.07% of ERα^POA^ neurons were activated by PPT, while 9.3% of ERα^POA^ neurons were inhibited by PPT, and 11.63% of ERα^POA^ neurons were unresponsive. The PPT-responsive subpopulations of ERα^POA^ neurons were not significantly changed in RE mice (Figure 6B,C and F). However, within PPT-activated ERα^POA^ neurons, the PPT-induced increase of firing frequency was significantly reduced in RE mice compared to those in virgin females, without changes in the resting membrane potential. RE changed neither the firing frequency nor the resting membrane potential reduced by PPT in PPT-inhibited ERα^POA^ neurons (Figure 6C–H). Together, these results suggested that RE reduced the excitatory effects of E2-ERα signaling on ERα^POA^ neurons.

## 3 Discussion

**Figure 7:**
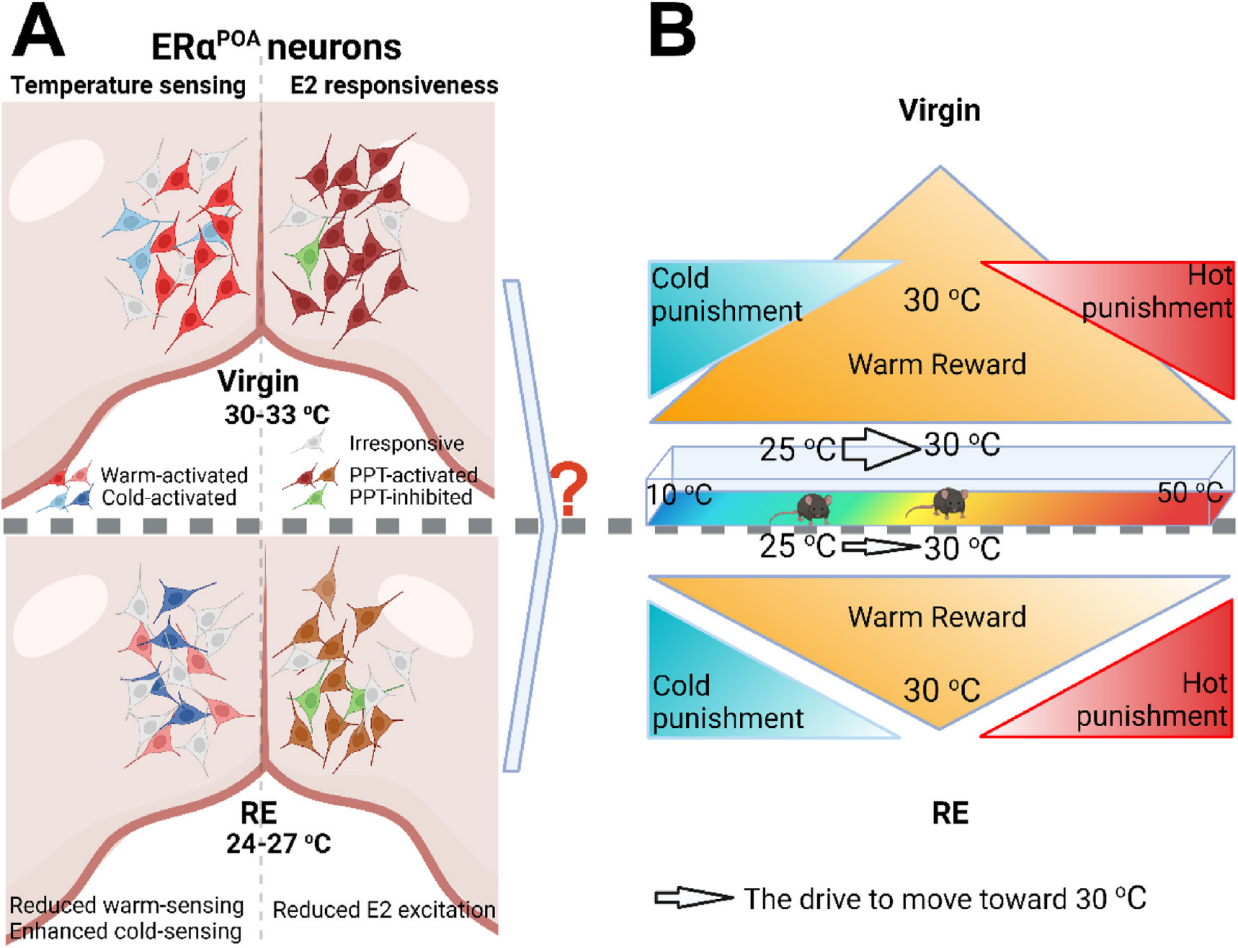
**Reproductive experience modulates thermal preference by altering temperature sensing and E2 responsiveness of ERα**^**POA**^**neurons.** (A) RE lowers thermal preferred temperature of female mice. Meanwhile, RE not only reduces warm-excitation ability and enhances cold-excitation ability of ERα^POA^ neurons, but also reduces their excitation by E2-ERα signaling. (B) A potential explanation of the lower preferred temperature in RE females. Negative feelings produced by cold or hotness (partially by cold- or hot- activated ERα^POA^ neurons) and warm reward (partially by warm-activated ERα^POA^ neurons) together drive mice to move toward their thermal preferred temperature zone, around 30 °C in virgin female mice (up). Impaired warm-excitation ability and reduced E2 excitation of ERα^POA^ neurons inhibited the warm reward in RE mice, so that RE mice lose the motivation to move toward warm environment from their habitual cooler environment (down).

Females experience a dramatic remodeling of the metabolic systems during reproduction, and some of the adaptations cause persistent or even permanent postpartum impacts. We found that RE mice display lower thermal preferred temperature and altered warmth-seeking behavior, associated with decreased number of ERα^POA^ neurons. Mechanistically, loss of ERα from POA neurons reduces thermal preferred temperature. In addition to the warm-excitation of ERα^POA^ neurons, we identified that a portion of ERα^POA^ neurons can be activated by temperature decrease both from the external environment and internally in the brain. Consistently, we identified that ERα^POA^ neurons are heterogeneous, with subpopulations of ERα^POA^ neurons being activated by either warm, or cold or neither. We found that RE impairs the warm-excitation but enhances the cold-excitation ability of ERα^POA^ neurons and reduces the excitatory effects of E2 on ERα^POA^ neurons. Together, the modulations of temperature-sensing and E2 responsiveness of ERα^POA^ neuron subpopulations may contribute to the impaired warm preference in RE mice (Figure 7A). However, it is unclear how it occurs. If ERα^POA^ neurons are required for thermal defensive behaviors, the reduced warm-excitation ability and enhanced cold-excitation ability of ERα^POA^ neurons should enhance warmth-seeking behavior (increase of thermal preferred temperature), which conflicts with our finding of the reduced thermal preferred temperature. One possible explanation is that ERα^POA^ subpopulations are required for the balance between the warm reward (by warm-activated ERα^POA^ subpopulation) and hot/cold punishment (a portion of warm-activated ERα^POA^ subpopulation and cold-activated ERα^POA^ subpopulation that sense extreme warm/cold or mediate warm/cold defensive behaviors), which drives virgin female mice to their preferred temperature around 30 °C. In this case, it would be reasonable that reduced warm-excitation ability of ERα^POA^ neurons decreases the warm reward that motivates RE mice to move toward the warm environment from their habitual 25 °C environment (Figure 7B).

### 3.1 Temperature-sensing in ERα^POA^ neurons regulates warm preference

For humans, extreme low (0–10 °C) or high (40 °C) temperatures are associated with negative feelings, and warmth is generally associated with positive valence [38]. Mechanistically, heat stimulus at lower ambient temperature can produce a rewarding feeling associated with positive valence. When the temperature increases above a threshold, e.g., 37 °C, this warmth sensing is not rewarding anymore. Thus, heat stimulus at a higher temperature can produce a punishing feeling associated with a negative valence [35]. Between cold and hot, temperature increase can activate warm-sensing neurons to produce a warm reward [[13], [14], [15]]. As evidenced, activation of warm-activated neurons in the POA produces a positive valence specifically at temperatures below thermoneutrality, potentially due to warmth reward [15]. The balance between the warm reward and negative feelings drives human and animals to move toward their thermal preferred temperature area. The POA is rich in warm-sensing neurons [14,15,[30], [31], [32], [33], [34]]. Consistent with a previous report [34], we found that half of ERα^POA^ neurons can be activated by warm temperatures, and some ERα^POA^ neurons are not responsive to temperature changes. Importantly, we found that both the population and the responsiveness of these warm-activated ERα^POA^ neurons are reduced in RE mice. Therefore, the impaired warm-excitation ability of these warm-activated ERα^POA^ neurons may reduce warm reward in a warming environment, which may explain the reduced warmth-seeking behavior and lower thermal preferred temperature in RE mice. However, we don’t know whether the reduced ERα^POA^ neurons by RE belong to the warm-activated ERα^POA^ population responsible for thermal preference regulation.

The reduced thermal preferred temperature in RE mice could not be explained by heat-defense/cold-seeking behavior generated by activation of warm-sensing neurons [[13], [14], [15]]. Reduced warm-excitation ability of these warm-activated ERα^POA^ neurons should reduce cold-seeking behavior but not the observed lower preferred temperature in RE mice. Lower thermal preferred temperature could also be a result of cold-defense/warmth-seeking behavior generated by activation of cold-sensing neurons. Consistent with the warmth-seeking behavior at a cold temperature (8 °C) [35], we showed that mice escaped from the negative feelings induced on the cold side (15 °C) and displayed warmth-seeking behavior by traveling to the 20 °C side. The coding of cold-defense/warmth-seeking behavior in a cold environment involves cold-sensing neurons [[13], [14], [15]]. The POA also contains cold sensing neurons [22,24,33]. In addition to the warm-activated ERα^POA^ neurons, we found that a small population of ERα^POA^ neurons can be activated by cold temperature, and these cold-activated ERα^POA^ neurons are enhanced in RE mice compared to virgin mice. If warmth-seeking behavior is coded by cold-activated ERα^POA^ neurons, RE would be expected to increase warmth-seeking behavior due to enhanced cold-excitation of ERα^POA^ neurons, which conflicts with the lower preferred temperature observed in RE mice. Therefore, neither heat-defense/cold-seeking behavior nor cold-defense/warmth-seeking behavior could explain the lower preferred temperature in RE mice.

### 3.2 Estrogenic effects in ERα^POA^ neurons regulate warm preference

E2 signaling, primarily mediated by ERα, is important for thermoregulation [42,[64], [65], [66], [67], [68]]. E2 signaling dramatically climbs during late pregnancy and then drops after delivery, which goes back to a normal level together with restoration of ovulation [[60], [61], [62]]. However, some of the E2 modulations in the central nervous system are not fully restored even after long-term postpartum recovery. Here we showed that the numbers of ERα neurons are reduced in the POA but not in the ARH or VMH 2 weeks after weaning. Interestingly, vehicle treated ovariectomized (OVX-V) rats prefer an environment 4 °C cooler than E2 treated ovariectomized (OVX-E) rats [64], supporting the function of E2 on the maintenance of warm preference. We found that virgin ERα^POA^-KO mice and RE mice with reduced ERα^POA^ neurons both lose preference for warm environments like OVX-V rats. These results suggest that the impaired warmth-seeking behavior could be attributed to the inhibited E2 signaling in the POA. While the majority of ERα^POA^ neurons can be activated by PPT, an ERα agonist, a small population of ERα^POA^ neurons can be inhibited by PPT. Despite unchanged subpopulations of PPT-activated or inhibited ERα^POA^ neurons, the excitatory effect of E2-ERα on PPT-activated ERα^POA^ neurons was attenuated in RE mice. Together, both the population of ERα^POA^ neurons and their excitation by E2-ERα signaling were significantly reduced in RE mice, and this suppressed E2-ERα signaling in ERα^POA^ neurons may lower thermal preferred temperature in RE mice. All pregnant, lactating and RE mice with varying E2 levels and virgin ERα^POA^-KO mice display the same altered thermal preference, which suggests that the RE effect is more about the modulation of ERα^POA^ neurons by reproduction or ERα than acute loss or gain of the ligand E2 in the POA. This may also explain why estrus mice just after the E2 peak of the proestrus phase preserve warm preference but diestrus mice with long-term low E2 incubation don’t, despite diestrus mice also having an E2 peak [46]. However, it is unclear whether RE decreases the expression of ERα or any downstream genes of ERα signaling per neuron in ERα^POA^ neurons to reduce their E2 excitation, or RE modulates the expression of the key molecules in ERα^POA^ neurons, e.g., thermosensors or neuron transmitters, to alter their warm- and cold-excitation.

E2 can also differentially regulate body temperature depending on the environmental temperature and innate status. Supplement of E2 centrally or injection of E2 into the VMH increases body temperature in OVX animals at room temperature [65]. However, oppositely, a subcutaneous supplement of E2 reduces body temperature in OVX rats in warmer environments (>30 °C) [64]. These results suggest that E2 is important for thermoregulation to maintain body temperature at a stable level. The estrogenic effects on thermoregulation are potentially mediated by the temperature-sensing ability of ERα neurons. Interestingly, body temperature can be increased by activation of ERα^vlVMH^ neurons [83] or other cold-sensing neurons in the VMH [37] but decreased by activation of ERα^POA^ neurons [34] or other warm-sensing neurons in the POA [15]. A previous report showed that ablation of ERα^POA^ neurons increases body temperature in female mice [34]. However, our data showed that body temperature is reduced in RE mice but not in ERα^POA^-KO mice, arguing the dispensable contribution of E2-ERα^POA^ signaling to the lower body temperature in RE mice. Notably, we showed that subpopulations of ERα^POA^ neurons can be activated or inhibited by E2-ERα signaling or temperature changes, or be unresponsive to these stimuli. Thus, the body temperature change in ERα^POA^-KO female mice is potentially due to the mixed impact of E2-ERα signaling on the three temperature-sensing ERα^POA^ neuron subpopulations. In addition, we observed impaired warm-excitation and enhanced cold-excitation in ERα^POA^ neurons of RE mice, which predict higher body temperature in RE mice if ERα^POA^ neuron subpopulations control body temperature. Therefore, our results do not support the contribution of E2-ERα signaling in ERα^POA^ neurons, as a whole population, to the lower body temperature in RE mice. This lower body temperature could be potentially explained by the modulation of other neuron populations, e.g., VMH neurons, since the VMH neurons are also modulated during reproduction [[84], [85], [86]] and involved in temperature-sensing and thermoregulation [37].

### 3.3 ERα^POA^ neurons sense temperature directly in the brain

Thermosensing neurons can sense temperatures indirectly through circuitry inputs from the peripheral nervous systems [[70], [71], [72], [73], [74]] or directly via thermosensors expressed in the neurons [33,69]. Our *in vivo* photometry recording data indicate that ERα^POA^ neurons can be regulated by ambient temperature. This acute temperature change should not be sufficient to change body or brain temperature, suggesting an indirect regulation of ERα^POA^ neurons via upstream inputs relayed from the peripheral thermosensors, e.g., in the skin. Long-term ambient temperature exposure can also change temperatures inside the brain, especially the POA [69]. We re-analyzed published scRNA-Seq data and found that ERα^POA^ neurons co-express many warm and cold sensors. These thermosensors may enable the intrinsic thermosensing ability of ERα^POA^ neurons.

Importantly, our *ex vivo* brain slice electrophysiology recording data indicate that ERα^POA^ neurons can also be regulated by temperature changes surrounding the neurons, with or without presynaptic blockers. These results support an intrinsic thermosensing ability of ERα^POA^ neurons independent of peripheral inputs. Together, ERα^POA^ neurons can be regulated by both the peripheral and internal temperature changes, but it is unclear whether these two mechanisms overlap or interplay.

Neurons are generally highly sensitive to temperature variations. Action potential conduction velocity increases with temperature, primarily due to the temperature dependence of ion channel kinetics. In particular, the activation and inactivation of voltage-gated sodium (Na⁺) and potassium (K⁺) channels are accelerated at higher temperatures [87]. Synaptic transmission, including presynaptic vesicle fusion and neurotransmitter release, is also accelerated at higher temperature due to an increase in the rate of biochemical reactions involved in vesicle docking and fusion [88]. Higher temperature also tends to enhance receptor sensitivity and conductance, thereby increasing postsynaptic current flow [89]. However, prolonged exposure to high temperatures can lead to receptor desensitization or dysfunction, impairing synaptic communication [90]. Thus, the warm- or cold-induced neuronal activity changes in ERα^POA^ neurons could also come from their presynaptic neurons other than the sensory neurons transmitted from the peripheral thermoreceptors. We found that presynaptic blockers did not change the responsiveness of ERα^POA^ neurons to temperatures in both virgin and RE mice, confirming the direct thermosensing ability of ERα^POA^ neurons independent of presynaptic inhibitory or excitatory inputs. Despite the expression profile of some warm-sensing markers like Trpc4 and Ptgds, and cold-sensing marker Cnga3 being changed by OVX-V vs OVX-E, it is unclear whether these neuron populations are also changed during reproduction.

### 3.4 Other contributors to the altered thermoregulations by RE

The preference for a cooler environment starts from late pregnancy and is enhanced during lactation, and then persists even after long-term postpartum. However, it is unclear when and how the ERα^POA^ neurons are modulated during pregnancy or lactation, and their contribution to temperature sensing during these stages. In addition to E2, progesterone also significantly increases during late pregnancy and then immediately drops after delivery, and prolactin significantly increases during late pregnancy and then surges during lactation in response to pups' suckling [[91], [92], [93]]. Receptors of these three hormones are highly co-expressed in many brain regions, including the POA [43,44]. Loss of prolactin receptor in the POA increased physical activity during pregnancy and impaired maternal behaviors [94]. Notably, prolactin suppresses thermogenesis during lactation [2,3,95,96] and a warm environment adversely impacts maternal behaviors [97,98]. Importantly, prolactin sensitivity in the hypothalamus was enhanced by RE [99], in alignment with the altered thermoregulation in RE mice. Thus, it is plausible that prolactin alters thermoregulations during late pregnancy and lactation. The levels or sensitivity of other metabolic hormones like leptin and insulin are also modulated during pregnancy and lactation [[100], [101], [102]]. Notably, LepR labels a population of warm-sensing neurons in the POA [77]. Thus, the contribution of these hormones cannot be excluded either.

Although the number of ERα neurons is not significantly reduced in the VMH and ARH by RE, it is unclear whether the activity of these ERα neurons or their responsiveness to E2 is modulated in RE mice. Further, we used PPT to estimate the rapid membrane-initiated responsiveness of ERα^POA^ neurons to E2-ERα signaling, which does not reflect the classic nuclear-initiated genetic effect of ERα and estrogenic effects mediated by other estrogen receptors, like ERβ, which is also co-expressed in a small population of ERα^POA^ neurons [44]. In addition, ERα is abundantly expressed in multiple brain regions including the lateral hypothalamus (LH), the nucleus of the solitary tract (NTS), the medial amygdala (MeA), and the dorsal raphe nuclei (DRN) [78,103]. These ERα neuron populations are also potentially modulated by reproduction due to fluctuating E2 or other reproductive hormones including progesterone, prolactin, oxytocin, etc [62,104,105]. It is also unclear whether other temperature-sensing neuron populations in the POA or in other hypothalamic regions, e.g., the LH [27,35], the DMH [36] or the VMH [37,[84], [85], [86]], are regulated by RE. The contribution of these ERα neuron populations and temperature sensing neuron populations also needs to be determined.

### 3.5 Limitations and remaining questions

We presume that a portion of the warm-activated ERα^POA^ subpopulation can produce positive valence. The reduced warm-excitation ability of the warm-activated ERα^POA^ subpopulation in RE mice reduces the warm reward produced in a warm environment, thus causing RE mice to lose the preference for warmth. However, we do not yet have data to support the assumption that warm-activated ERα^POA^ subpopulation does produce a positive valence.

Our current studies could not distinguish the functions of the temperature-sensing and/or estrogen-ERα-responsive subpopulations of ERα^POA^ neurons on thermoneutral temperature, body temperature and valence. Since activation of ERα^POA^ neurons reduces both body temperature and thermal preferred temperature of female mice, one question is whether the reduced thermal preferred temperature is a consequence of reduced body temperature, or whether they are regulated by the same mechanism. Our data showed that decreased body temperature and preferred temperature co-exist in RE mice but not in lactating mice or virgin ERα^POA^-KO mice, which suggests that body temperature and thermal preferred temperature are not bundled. Our study deleted the ERα gene from most ERα^POA^ neurons, regardless of whether they are activated or inhibited by E2-ERα or temperature changes. It is unclear how E2 regulates these three subpopulations of ERα^POA^ neurons and whether their modulation contributes to the impaired warmth-seeking behavior in RE mice. Therefore, body temperature, thermal preferred temperature, and valence may be regulated by distinct mechanisms in different subpopulations of ERα^POA^ neurons, which needs further investigation.

We noticed a negative correlation between physical activity and warm preference in virgin female mice. It is unclear whether high physical activity drives cold-seeking behavior via increasing body temperature, or female mice with low physical activity lack exploration motivation and stay in thermoneutral areas. The majority of G16 dams reduced physical activity, potentially due to heavier body weight or the burden of pups. Only two G16 outliers display high physical activity and still prefer to stay in warm areas. However, physical activity is significantly increased during lactation and then recovers in RE mice, and loss of the ERα from ERα^POA^ neurons does not change physical activity. It is unclear whether the lactation-related high physical activity is related to stress or anxiety induced by pup separation or thermal sensing changes. Thus, physical activity and thermal preference are likely regulated by separate mechanisms.

The AAV-Virus strategy is another technical limitation. Viruses may not reliably target all neuron populations in all POA areas due to variations induced by injection and virus diffusion per se. Thus, future studies should be considered to refine the subpopulation of ERα^POA^ neurons in specific POA subareas.

We acknowledge that body temperature varies with circadian and estrous phases [46], and that only one or two time points daily are not adequate for reproductive mice and ERα^POA^-KO mice. Similarly, the activity of each ERα^POA^ subpopulation may also fluctuate. A photometry measurement of each subpopulation and a 24-hour temperature profile across different estrous phases and reproductive stages are recommended for a more comprehensive analysis in future studies.

Finally, we used an electrophysiology recording protocol by changing bath temperature between 25 °C and 30 °C or 30–34 °C, which do not represent real internal brain temperature range 33–39 °C. Regardless, we provide a proof-of-concept that ERα^POA^ neurons are regulated by temperature changes from both the external environment and internally in the brain. Our results imply that the temperature changes in the external environment and internal brain may merge at some points, resulting in many new questions to be explored in the future. E.g., how much is the overlay? Does it apply to all temperature sensing neurons or all brain regions? How do these neurons integrate the responses from the external environment and the internal brain?

## 4 Materials and methods

### 4.1 Mice

Care of all animals and procedures were approved by the Institutional Animal Care and Use Committee of Baylor College of Medicine and Pennington Biomedical Research Center. Mice were housed in a temperature-controlled room at 22–24 °C using a 12-h light, 12-h dark cycle. To exclude the effects of diet and age that interfere with the results, we used age-matched littermates to generate all the control and experimental mice and fed them with the same diet. All the mice including control virgin female mice were fed a breeding diet (5V5M, PicoLab) when RE mice were pregnant and lactating. All these mice were fed with regular chow (5V5R, PicoLab) during the periods before pregnancy and after the weaning of pups for RE mice. Water was provided ad libitum.

### 4.2 Generation of pregnant, lactating and RE mice and age-matched virgin female mice

8–10 weeks of age C57BL/6 background wild type female mice were implanted with a telemetry probe (IPTT-300, BioMedic Data Systems) underneath the skin to measure body temperature. All the mice were randomized into two groups. One group were bred with male mice to generate pregnant mice, lactating mice and RE mice. The other group served as age-matched littermate controls. These female mice were subjected to thermogradient box test on the 16th day of gestation (G16), the 6th day of lactation (PPD6) and 1 months after pups weaning (PW4w). PPD6 dams were separated from the pups just prior to the test. Briefly, mice were placed in a thermal gradient test box (Bio-TGT2, Bioseb) with one side set to 10 °C and the other side set to 50 °C for 30 min habituation followed by 30 min recording. The time spent in temperature zones was recorded and analyzed using Thermal Gradient Test software (Bioseb). The preferred temperature is the zone temperature in which mice spent longest time throughout the whole recording. On the day of thermogradient box test on G16, vaginal cells of the virgin female mice were flushed and observed under a light microscope to estimate estrous phases based on the proportion of leucocytes, cornified epithelial cells and nucleated epithelial cells.

Another batch of singly caged RE mice (5–7 months after weaning of pups) were used for the behavior tests as described below, and their body temperatures were measured at 10 am using a thermometer probe (DIGI-Sense, Type J/K/T, thermocouple meter, WD-20250-91).

### 4.3 Deletion of ERα from POA neurons

Female ERα^flox/flox^ mice [106] (10-week) were anesthetized by isoflurane and received bilateral stereotaxic injections of AAV-Cre-GFP or AAV-GFP virus (200 nl, 5∗10^12^ GC/ml, UNC Gene Therapy Center) into the POA (ML +/−0.30, AP 0.48 mm, DV -5.40 mm). Meanwhile, all the mice were implanted with temperature probes (IPTT-300, BioMedic Data Systems) underneath the skin of the back to monitor body temperature. All the mice were single caged for the measurements of body weight, food intake and body temperature at 10 am or 5pm. 5 weeks after virus injection, these mice were subject to thermogradient box test.

### 4.4 Generation of ERα^POA^-GCaMP6

Virgin female ERα-Cre transgenic mice [107] (10-week) were anesthetized by isoflurane and received bilateral stereotaxic injections of AAV-DIO-GCaMP6 virus (200 nl, 5∗10^12^ GC/ml, Addgene, #100838) into the POA (ML 0.30, AP 0.48 mm, DV -5.40 mm) to express GCaMP6 protein specifically in ERα^POA^ neurons (ERα^POA^-GCaMP6). During the same surgery, an optic fiber (200 μm core, R–FOC–BL200C-39NA, RWD) was placed over the POA (ML 0.30, AP 0.48 mm, DV -5.15 mm) of ERα^POA^-GCaMP6 mice to record fluorescence of GCaMP6. These mice were fed with chow diet and recovered for at least two weeks before any other procedures.

### 4.5 Behavior tests

Mice were acclimated to a homecage-like cage test box without bedding for 10 min on the day before the experiment. For warm preference test, half side of the test box was preheated with a heat mat under the bottom for 5 min to reach and then maintain a stable temperature around 30 °C during the test, with the other side unheated around 25 °C. For cold avoidance test, half side of the test box was submerged in ice for 5 min to reach and then maintain a stable temperature around 15 °C during the test, with the other side unheated around 20 °C. Mice were put gently in the middle line of the cage and were allowed to explore the two sides freely for 5 min. The procedures were videotaped and analyzed using an EthoVision XT (Version 14.0, Noldus Information Technology BV) software. Staying time, travel distance and velocity in each side were calculated for each mouse. 18-20

### 4.6 Fiber photometry

Activity of ERα^POA^ neurons were recorded using fiber photometry strategy. One day prior to recording, mice were given 10 min to acclimate to the tethered patchcord. To record the response of ERα^POA^ neurons to changing temperature, ERα^POA^-GCaMP6 mice were allowed to explore the box which was used for warm preference test or cold avoidance test. Continuous <20 μW blue LED at 465 nm and UV LED at 405 nm were served as excitation light sources, driven by a multichannel hub (Doric Lenses), modulated at 211 Hz and 330 Hz, respectively. The light was delivered to a filtered minicube (FMC5, Doric Lenses) before connecting through optic fibers to a rotary joint (FRJ 1 × 1, Doric Lenses) to allow for movement. GCaMP6 calcium GFP signals and UV autofluorescent signals were collected through the same fibers back to the dichroic ports of the minicube into a femtowatt silicon photoreceiver (2151, Newport). The digital signals were then amplified, demodulated, and collected through a lock-in amplifier (RZ5P, Tucker–Davis Technologies). The fiber photometry data were collected using Synapse 2.0 (Tucker–Davis Technologies) and down sampled to 8 Hz. To calculate ΔF/F, a least-squares linear fit was applied to the 405 nm signal to align it to the 465 nm signal, producing a fitted 405 nm signal that was used to normalize the 490 nm as follows: ΔF/F = (465 nm signal − fitted 405 nm signal)/fitted 405 nm signal [108]. Time 0 is defined as moment when mice crossed the line of the two sides with different temperatures, i.e., 25 °C → 30 °C or 20 °C → 15 °C.

### 4.7 Immunohistochemistry and H&E staining

RE mice were perfused 6–12 days after the weaning of pups at fed and diestrus phase. ERα^POA^-KO mice were perfused after thermogradient box test. Brains were processed for frozen sectioning at 25 μm and collected into 5 separate series. One series of brain sections were subjected to post hoc histological validation of virus injection accuracy. Another series of brain sections were processed for immunohistochemistry staining for ERα. Briefly, brain sections were blocked (3% Normal donkey serum) for 1 h, incubated with Rabbit anti-ERα Antibody (06–935, Millipore) on shaker at 4 °C for overnight, followed by the donkey anti-rabbit AlexaFluor 488 (2376850, Invitrogen) for 2 h. Slides were cover-slipped and images were captured using a fluorescence microscope with 1-second exposure time and no change of contrast. Images of ARH, VMH and POA were captured from rostral to caudal consecutively. The number of ERα neurons were calculated by ImageJ (NIH) and counted from one side of ARH for 7 slices, VMH for 4 slices and POA for 5 slices in each mouse.

### 4.8 Electrophysiological recordings

8-week ERα-ZsGreen [78] virgin female mice were used to generate RE mice. One month after the weaning of pups, RE mice and age matched ERα-ZsGreen virgin mice (in diestrus) were used for electrophysiological recordings.

Mice were deeply anesthetized with isoflurane and transcardially perfused with an ice-cold sucrose-based cutting solution (pH 7.3) containing 10 mM NaCl, 25 mM NaHCO3, 195 mM sucrose, 5 mM glucose, 2.5 mM KCl, 1.25 mM NaH2PO4, 2 mM Na-pyruvate, 0.5 mM CaCl2, and 7 mM MgCl2 bubbled continuously with 95% O2 and 5% CO2 [[109], [110], [111], [112]]. High concentration of sucrose in the cutting solution will protect neurons from damage during the slice cutting. The mice were then decapitated, and the entire brain was removed and immediately submerged in cutting solution. Slices (250 μm) were cut with a Leica VT 1200s (Leica Biosystems). Three to four random brain slices containing the POA (bregma −0.58 mm to +0.5 mm) were obtained for each mouse. The slices were recovered for 1 h at 34 °C and then maintained at room temperature in artificial cerebrospinal fluid (aCSF, pH 7.3) containing 126 mM NaCl, 2.5 mM KCl, 2.4 mM CaCl2, 1.2 mM NaH2PO4, 1.2 mM MgCl2, 5.0 mM glucose, and 21.4 mM NaHCO3 saturated with 95% O2 and 5% CO2 before recording.

Slices were transferred to a recording chamber and allowed to equilibrate for at least 10 min before recording. The slices were superfused at 34 °C in oxygenated aCSF at a flow rate of 1.8–2 ml/min. In the POA, ZsGreen-labeled neurons were visualized using epifluorescence and IR-DIC imaging on an upright microscope (Eclipse FN-1, Nikon) equipped with a movable stage (MP-285, Sutter Instrument). Patch pipettes with resistances of 3–5 MΩ were filled with intracellular solution (pH 7.3) containing 128 mM K-gluconate, 10 mM KCl, 10 mM HEPES, 0.1 mM EGTA, 2 mM MgCl2, 0.05 mM Na-GTP, and 0.05 mM Mg-ATP. Recordings were made using a MultiClamp 700B amplifier (Axon Instrument), sampled using Digidata 1440 A, and analyzed offline with pClamp 10.3 software (Axon Instruments). Series resistance was monitored during the recording, and the values were generally <10 MΩ and were not compensated. The liquid junction potential was +12.5 mV and was corrected after the experiment. Data were excluded if the series resistance increased dramatically during the experiment or without overshoot for the action potential. Currents were amplified, filtered at 1 kHz, and digitized at 10 kHz. The current clamp was engaged to test neural firing frequency and resting membrane potential with or without PPT (100 nM, 5 s puff treatment) [82,83]. For temperature sensing experiments, recorded POA ERα neurons were challenged by temperature changes: protocol 30 °C→ 34 °C→30 °C for TRAP2/ERα-Flpo mice, and protocol 25 °C→30 °C→25 °C [34] for RE mice. The pH of aCSF was not significantly altered with continuous CO2 bubbling at 30 and 34 °C. To further test if temperature directly changed neuronal firing activity, cocktail synaptic blockers (30 μM CNQX, 30 μM D-AP5, and 50 μM bicuculline) were added to the bath solution. The values for resting membrane potential and firing frequency averaged within a 1-min bin. Any neuron without spontaneous firing was excluded from the analysis of firing frequency analysis but was included in the analysis of resting membrane potential. A neuron was considered activated or inhibited with >20% increases or decreases in firing frequency or at least 2 mV increases or decreases in amplitude.

### 4.9 Secondary analysis of single cell RNA-Seq data

scRNA-seq data were obtained from GSE113576 (Moffitt et al., 2018) [43] and GSE183093 (Knoedler et al., 2022) [44]. Data were initially analyzed using Seurat 4.3.0 in R 4.3.2. Respective authors' annotations were used to isolate neurons and experimental groups (i.e. males, females, OVX females + vehicle, and OVX females + E2). Custom functions were written to query the Seurat objects for co-expression of Esr1 and a list of target genes utilizing functions from Seurat and dplyr 1.1.3. These data were then visualized using ggplot2 3.4.4.

### 4.10 Statistical analyses

The data are presented as mean ± SEM and/or individual points. Statistical analyses were performed using GraphPad Prism to evaluate normal distribution and variations within and among groups. Methods of statistical analyses were chosen based on the design of each experiment and are indicated in Figure legends. *P* < 0.05 was considered to be statistically significant.

## Funding

Investigators involved in this work were supported by grants from the 10.13039/100000199USDA/CRIS (3092-51000-062-04(B)S to C.W), 10.13039/100020951PBRC institutional funding to Y.H, 10.13039/501100001809National Natural Science Foundation of China (82200927 to N.Z, 82270909 to T.Z.).

## Consent statement

None.

## CRediT authorship contribution statement

**Nan Zhang:** Validation, Software, Methodology, Investigation, Funding acquisition, Data curation, Conceptualization. **Meng Yu:** Validation, Methodology, Investigation, Formal analysis, Data curation. **Qianru Zhao:** Validation, Methodology, Investigation, Formal analysis, Data curation. **Bing Feng:** Methodology, Formal analysis, Data curation. **Yue Deng:** Investigation, Data curation. **Jonathan C. Bean:** Formal analysis, Data curation. **Qingzhuo Liu:** Methodology, Formal analysis, Data curation. **Benjamin P. Eappen:** Investigation, Data curation. **Yang He:** Methodology, Data curation. **Kristine M. Conde:** Methodology, Data curation. **Hailan Liu:** Methodology, Data curation. **Yongjie Yang:** Methodology, Data curation. **Longlong Tu:** Methodology, Data curation. **Mengjie Wang:** Methodology, Data curation. **Yongxiang Li:** Methodology, Data curation. **Na Yin:** Methodology, Data curation. **Hesong Liu:** Methodology, Data curation. **Junying Han:** Methodology, Data curation. **Darah Ave Threat:** Methodology, Data curation. **Nathan Xu:** Methodology, Data curation. **Taylor Smiley:** Methodology, Data curation. **Pingwen Xu:** Writing – review & editing, Conceptualization. **Lulu Chen:** Writing – review & editing, Conceptualization. **Tianshu Zeng:** Writing – review & editing, Writing – original draft, Supervision, Resources, Funding acquisition, Conceptualization. **Yanlin He:** Writing – review & editing, Writing – original draft, Visualization, Supervision, Funding acquisition, Formal analysis, Data curation, Conceptualization. **Chunmei Wang:** Writing – review & editing, Writing – original draft, Visualization, Supervision, Resources, Investigation, Funding acquisition, Formal analysis, Data curation, Conceptualization.

## Declaration of competing interest

The authors declare that they have no known competing financial interests or personal relationships that could have appeared to influence the work reported in this paper.

## Figures and Tables

**Figure:**
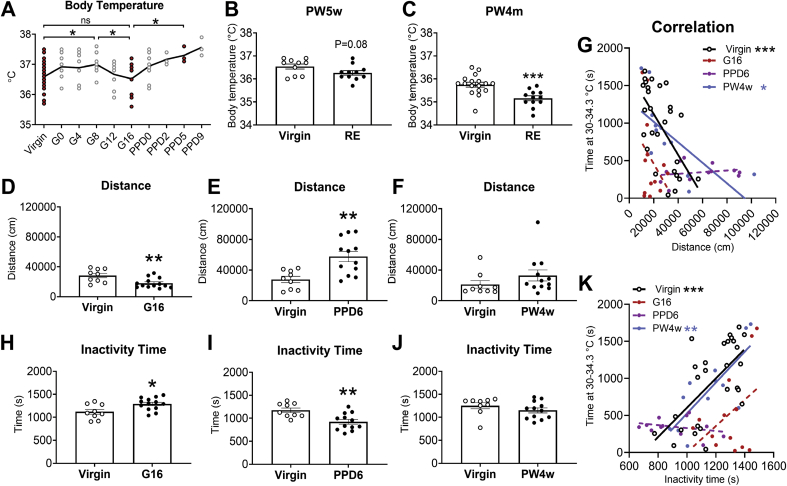
**Supplemental Fig. 1** (A) Body temperature of female mice at different stage of reproduction. (B–C) Body temperatures of 5 weeks post-weaning RE mice (B) and 4 months post-weaning RE mice (C) and their age-matched virgin female mice. Data are presented as mean ± SEM or with individual data points. ∗, *P* < 0.05 in one-way ANOVA analysis followed by Sidak’s test (A), *N* = 3–39. ∗∗∗, *P* < 0.001 in two-tailed unpaired t-tests (B–C), *N* = 9–17. (D-F) The distance that dams at G16 (D), PPD6 (E) and PW4w (F) and their age-matched virgin female mice traveled in a thermogradient box. (G) The correlation between time spent in the 30–34.3 °C and travelling distance. (H-J) The in active time that dams at G16 (H), PPD6 (I) and PW4w (J) and their age-matched virgin female mice spent in a thermogradient box. (G) The correlation between time spent in the 30–34.3 °C and in active time. Data are presented as mean ± SEM or with individual data points. ∗ and ∗∗, *P* < 0.05 or 0.01 in two-tailed unpaired t-tests (D, E, H and I). ∗, ∗∗and ∗∗∗, *P* < 0.05, 0.01 or 0.001 in Pearson correlation analysis (G and K). *N* = 9–13.

**Figure:**
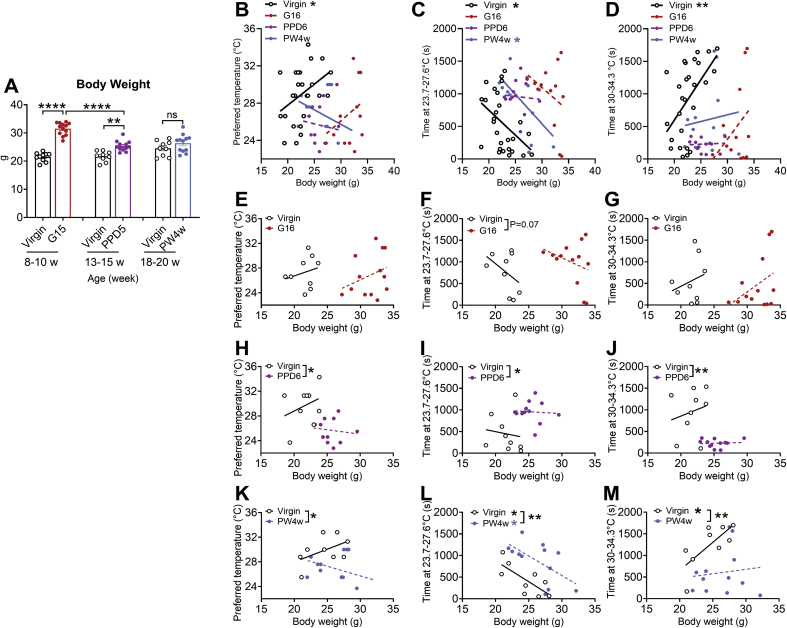
**Supplemental Fig. 2** (A) Body weight of G16, PPD6, PW4w mice and their age-matched virgin female mice. (B-D) The correlation between body weight and preferred temperature (B), time spent in the 30–34.3 °C (C) or 23.7–27.6 °C (D). (E-M) The correlation between body weight and preferred temperature, time spent in the 30–34.3 °C or 23.7–27.6 °C in G16 (E-G), PPD6 (H-J) and PW4w (K-M) mice with their age-matched virgin female mice. Data are presented as mean ± SEM or with individual data points. ∗∗ and ∗∗∗∗, *P* < 0.01 or 0.0001 in One-way ANOVA followed by Sidak test (A). ∗ and ∗∗, *P* < 0.05 or 0.01 in Pearson correlation analysis (B, C, D, L and M) and linear regression test (H-M). *N* = 9–13.

**Figure:**
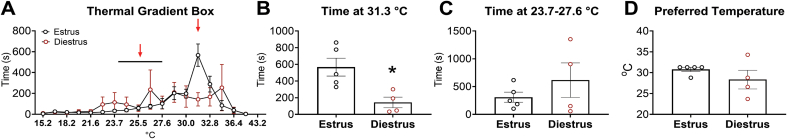
**Supplemental Fig. 3** (A–C) Time that estrus and diestrus virgin female mice spent in a thermogradient box (A), and at 31.3 °C (B) or 23.7–27.6 °C (C). (D) The preferred temperature that estrus and diestrus virgin female mice spent in a thermogradient box. Red arrows (J) indicate the areas displaying the most dramatic differences. Data are presented as mean ± SEM or with individual data points. ∗, *P* < 0.05 in two-tailed unpaired t-tests, *N* = 4–5.

**Figure:**
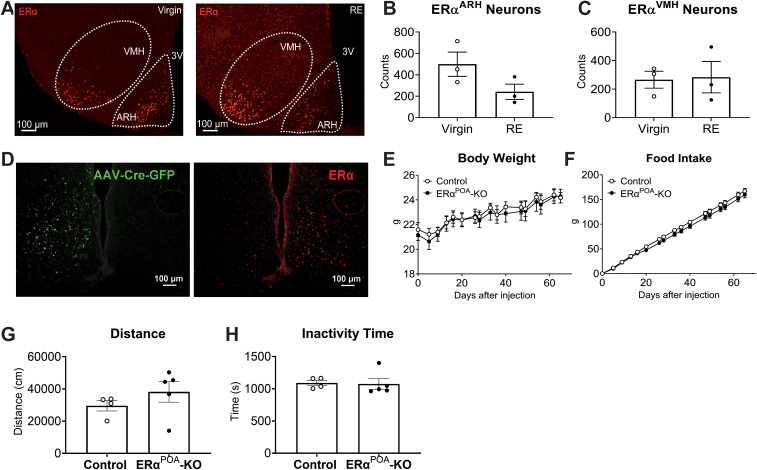
**Supplemental Fig. 4** (A–C) Immunostaining of ERα (A) and quantification ERα neurons (B–C) in the ARH and vlVMH of RE (6–12 days after weaning) mice and age-matched virgin female mice. Data are presented as mean ± SEM or with individual data points. ∗, *P* < 0.05 in two-tailed unpaired t-tests. *N* = 3. (D) Immunostaining of ERα in the POA of virgin ERα^POA^-KO mice, with left side hit with AAV-Cre-GFP virus and the right side missed. (E-F) Body weight (E) and accumulated food intake (F) of virgin ERα^POA^-KO mice and control female mice. *N* = 4–5. (G-H) The travelling distance (G) and inactivity time (H) that control mice and virgin ERα^POA^-KO mice spent in a thermogradient box.

**Figure:**
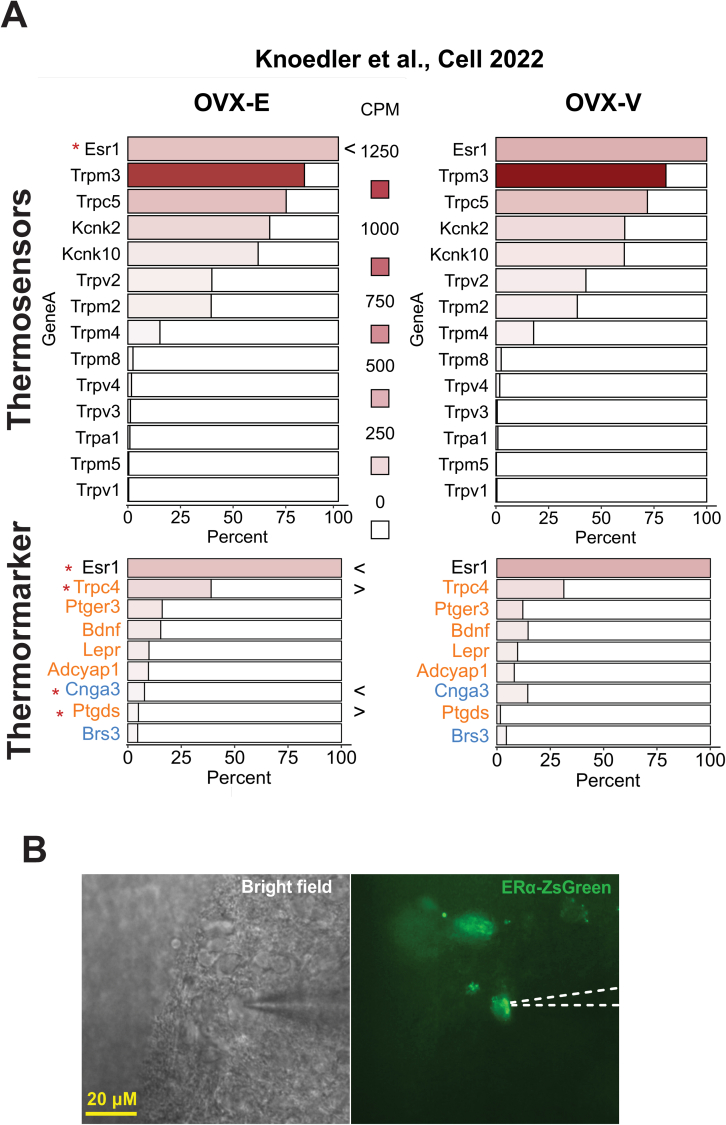
**Supplemental Fig. 5** (A) The expression of genes encoding thermosensors and thermomarkers in ERα^POA^ neurons in OVX-E vs OVX-V mice by a secondary analysis of published scRNA-Seq data [44]. CPM, counts per million. The color intensity indicates the abundance of each gene in ERα^POA^ neurons, while the width of the bar represents the percent expressing of each gene. ∗, *P* < 0.05 in Wilcox test followed by Bonferroni correction for multiple comparisons via Seurat 5.1.0. (B) Bright-field illumination (left) and fluorescence for ZcGreen (right) of a recorded ERα^POA^ neuron in a brain slice.

**Figure:**
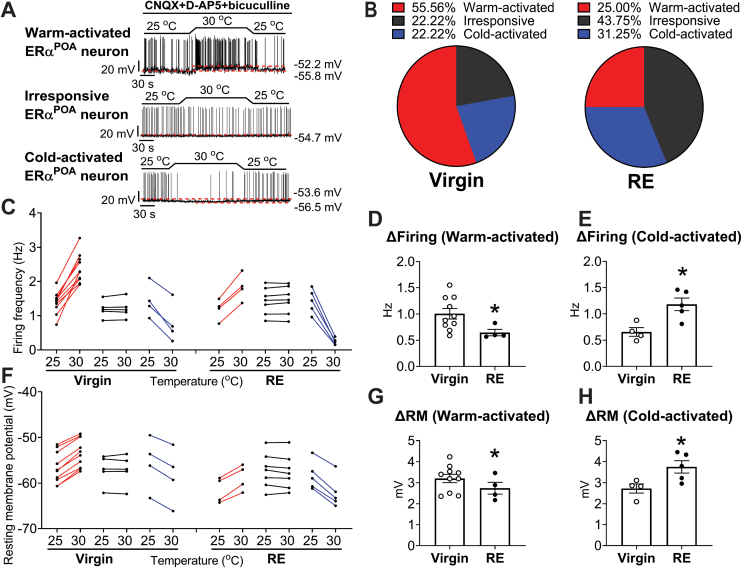
**Supplemental Fig. 6** Presynaptic blockers did not change the temperature sensing ability of ERα^POA^ neurons. (A) Representative traces of a warm-activated (up), an irresponsive (middle) or and a cold-activated (down) ERα^POA^ neurons by temperature increasing with presynaptic blocker (30 μM CNQX, 30 μM D-AP5, and 50 μM bicuculline). Warm activation or inhibition was defined as either >20% increases or decreases in firing rate or by > 2 mV depolarization or hyperpolarization. (B) Percentage of warm-activated, irresponsive or cold-activated ERα^POA^ neurons in age-matched virgin and RE female mice with presynaptic blocker. (C-E) Spontaneous firing rate (C), increases of firing rate in warm-activated ERα^POA^ neurons when temperature went up from 25 °C to 30 °C (D) and in cold-activated ERα^POA^ neurons when temperature went down from 30 °C to 25 °C (E) with presynaptic blocker. (F–H) Resting membrane potential (F), depolarization in warm-activated ERα^POA^ neurons when temperature went up from 25 °C to 30 °C (G) and in cold-activated ERα^POA^ neurons when temperature went down from 30 °C to 25 °C (H) with presynaptic blocker. Data are presented as part of whole bar graphs (B) or mean ± SEM and/or individual data points (C–H). ∗, *P* < 0.05 in two-tailed unpaired t-tests Virgin vs RE. *N* = 4–10 neurons from 3 mice/group.

## Data Availability

Data will be made available on request.
